# Database Resources of the National Genomics Data Center, China National Center for Bioinformation in 2025

**DOI:** 10.1093/nar/gkae978

**Published:** 2024-11-11

**Authors:** CNCB-NGDC Members and Partners, Yiming Bao, Xue Bai, Congfan Bu, Haobin Chen, Huanxin Chen, Kunqi Chen, Meili Chen, Miaomiao Chen, Ming Chen, Ping Chen, Qiancheng Chen, Qiaoshuang Chen, Runsheng Chen, Tingting Chen, Tong Chen, Xu Chen, Wenzhuo Cheng, Ying Cui, Mengting Ding, Lili Dong, Guangya Duan, Zhuojing Fan, Lu Fang, Zihao Feng, Shanshan Fu, Feng Gao, Ge Gao, Hao Gao, Suwei Gao, Xin Gao, Jing Gong, Yujie Gou, Anyuan Guo, Guoji Guo, Cheng Han, Fengxian Han, Zhenxian Han, Shunmin He, Daiyun Huang, Jinyan Huang, Xinhe Huang, Huijing Jiang, Jie Jiang, Shuai Jiang, Shuxian Jiang, Tao Jiang, Enhui Jin, Weiwei Jin, Hailong Kan, Zhixin Kang, Demian Kong, Ming Lei, Chuanyun Li, Cuiping Li, Hao Li, Jiang Li, Jing Li, Liuyang Li, Lun Li, Qiang Li, Rujiao Li, Xia Li, Xuan Li, Yixue Li, Yizhuo Li, Zhao Li, Chengzhi Liang, Yunchao Ling, Bo Liu, Chunjie Liu, Dan Liu, Feng Liu, Guanghui Liu, Haochen Liu, Lei Liu, Lin Liu, Mengyao Liu, Wan Liu, Wei Liu, Yanhu Liu, Yucheng Liu, Xuemei Lu, Hao Luo, Mei Luo, XiaoTong Luo, Zheng Luo, Jiongming Ma, Lina Ma, Shuai Ma, Yingke Ma, Jialin Mai, Jia Meng, Xianwen Meng, Yuyan Meng, Yaru Miao, Zepu Miao, Zhi Nie, Xiaohui Niu, Bing Pei, Di Peng, Jianzhen Peng, Juntian Qi, Yue Qi, Qiheng Qian, Qin Qiao, Jing Qu, Jian Ren, Zhengqi Sang, Yunfei Shang, Wenkang Shen, Yanting Shen, Han Shi, Meilong Shi, Wenwen Shi, Bowen Song, Shuhui Song, Jiani Sun, Yanling Sun, Yubin Sun, Bixia Tang, Dachao Tang, Qing Tang, Dongmei Tian, Zhixi Tian, Anke Wang, Fengping Wang, Fengyu Wang, Guodong Wang, Jianxin Wang, Lu Wang, Miaomiao Wang, Shiting Wang, Si Wang, Xiaohan Wang, Xuan Wang, Yanan Wang, Yanqing Wang, Yi Wang, Yibo Wang, Yinzhao Wang, Yonggang Wang, Zefeng Wang, Yaoke Wei, Zhen Wei, Dingfeng Wu, Song Wu, Wenyi Wu, Xueting Wu, Zishan Wu, Jingfa Xiao, Leming Xiao, Yun Xiao, Gui-Yan Xie, Guiyan Xie, Yubin Xie, Zhuang Xiong, Chenle Xu, Lingyun Xu, Ping Xu, Tianyi Xu, Ruikun Xue, Yu Xue, Chenyu Yang, Dechang Yang, Fei Yang, Jian Yang, Jiaxin Yang, Kuan Yang, Liu Yang, Xiaoyu Yang, Yuntian Yang, Haokai Ye, Caixia Yu, Chunhui Yuan, Hao Yuan, Liyun Yuan, Yuan Yuan, Jiaxing Yue, Shuang Zhai, Chi Zhang, Di Zhang, Guoqing Zhang, Jinyang Zhang, Mochen Zhang, Qiong Zhang, Shan Zhang, Shaosen Zhang, Sisi Zhang, Weiqi Zhang, Xiaolong Zhang, Xin Zhang, Yadong Zhang, Yang Zhang, Yaping Zhang, Yifan Zhang, Yiran Zhang, Yong E Zhang, Yongqing Zhang, Yuxin Zhang, Zhang Zhang, Fangqing Zhao, Guoping Zhao, Jing Zhao, Miaoying Zhao, Wei Zhao, Wenming Zhao, Xuetong Zhao, Yilin Zhao, Zheng Zhao, Xinchang Zheng, Xing Zheng, Bowen Zhou, Chenfen Zhou, Hanwen Zhou, Xinyu Zhou, Yubo Zhou, Junwei Zhu, Ruixin Zhu, Tongtong Zhu, Yan Zhu, Xinhao Zhuang, Wenting Zong, Dong Zou, Chunman Zuo, Zhixiang Zuo

## Abstract

The National Genomics Data Center (NGDC), which is a part of the China National Center for Bioinformation (CNCB), offers a comprehensive suite of database resources to support the global scientific community. Amidst the unprecedented accumulation of multi-omics data, CNCB-NGDC is committed to continually evolving and updating its core database resources through big data archiving, integrative analysis and value-added curation. Over the past year, CNCB-NGDC has expanded its collaborations with international databases and established new subcenters focusing on biodiversity, traditional Chinese medicine and tumor genetics. Substantial efforts have been made toward encompassing a broad spectrum of multi-omics data, developing innovative resources and enhancing existing resources. Notably, new resources have been developed for single-cell omics (scTWAS Atlas), genome and variation (VDGE), health and disease (CVD Atlas, CPMKG, Immunosenescence Inventory, HemAtlas, Cyclicpepedia, IDeAS), biodiversity and biosynthesis (RefMetaPlant, MASH-Ocean) and research tools (CCLHunter). All resources and services are publicly accessible at https://ngdc.cncb.ac.cn.

## Graphical Abstract

**Graphical Abstract:**
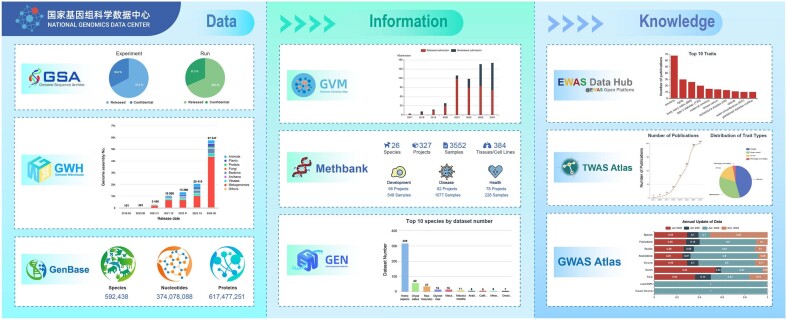

## Introduction

The National Genomics Data Center (NGDC), established in 2019, is affiliated with the China National Center for Bioinformation (CNCB), Beijing Institute of Genomics (BIG) and the Chinese Academy of Sciences (CAS) (1). CNCB-NGDC, in collaboration with the Institute of Biophysics and the Shanghai Institute of Nutrition and Health of CAS, has built strategic partnerships with numerous organizations (https://ngdc.cncb.ac.cn/partners) throughout the country. Particularly, in the last year, three subcenters have been established (https://ngdc.cncb.ac.cn/subcenter), including the Subcenter of Biodiversity (NGDC-BDV) in Kunming, the Subcenter of Traditional Chinese Medicine (NGDC-TCM) in Beijing and the Subcenter of Tumor Gene Diagnosis Data (NGDC-TGD) in Hangzhou. NGDC-BDV, hosted by the Kunming Institute of Zoology, CAS, focuses on biodiversity data across ecological, species and genetic dimensions. It oversees 1.5 billion pieces of scientific data and manages key databases like the Biodiversity Big Data Platform and the China Dragonfly Network, advancing global biodiversity research and conservation. NGDC-TCM, supported by the China Academy of Chinese Medical Sciences, aims to standardize and advance scientific data resources for TCM and integrate proteomic, metabolomic and transcriptomic data from TCM samples and medicinal plants. NGDC-TGD, maintained by the Biomedical Big Data Center at the First Affiliated Hospital of Zhejiang University School of Medicine, focuses on aggregating and managing tumor genetic data to address clinical challenges and improve cancer diagnostics.

Recent advancements in high-throughput sequencing technologies have propelled biological research into a multi-omics era, enriched by single-cell and spatial omics approaches (2,3). Large-scale initiatives such as Human Cell Atlas (4), Earth BioGenome Project (5), Single-Cell Expression Atlas (6), UK Biobank (7) and ImmPort (8) have produced extensive datasets encompassing genomics, transcriptomics, epigenomics, proteomics, immunomics, metabolomics, single-cell omics and spatial omics. These multidimensional, high-resolution datasets comprehensively characterize biological systems, including detailed cellular maps, cellular interactions and immune microenvironments. Through these datasets, researchers can explore developmental processes (9), immune responses (10,11), aging mechanisms (12,13), disease etiology (14,15) and potential therapeutic targets from multiple angles, accordingly providing critical insights into the genetic foundations of diseases and precision medicine applications, and advancing our understanding of complex cellular functions and biological processes (16).

**Figure 1.**
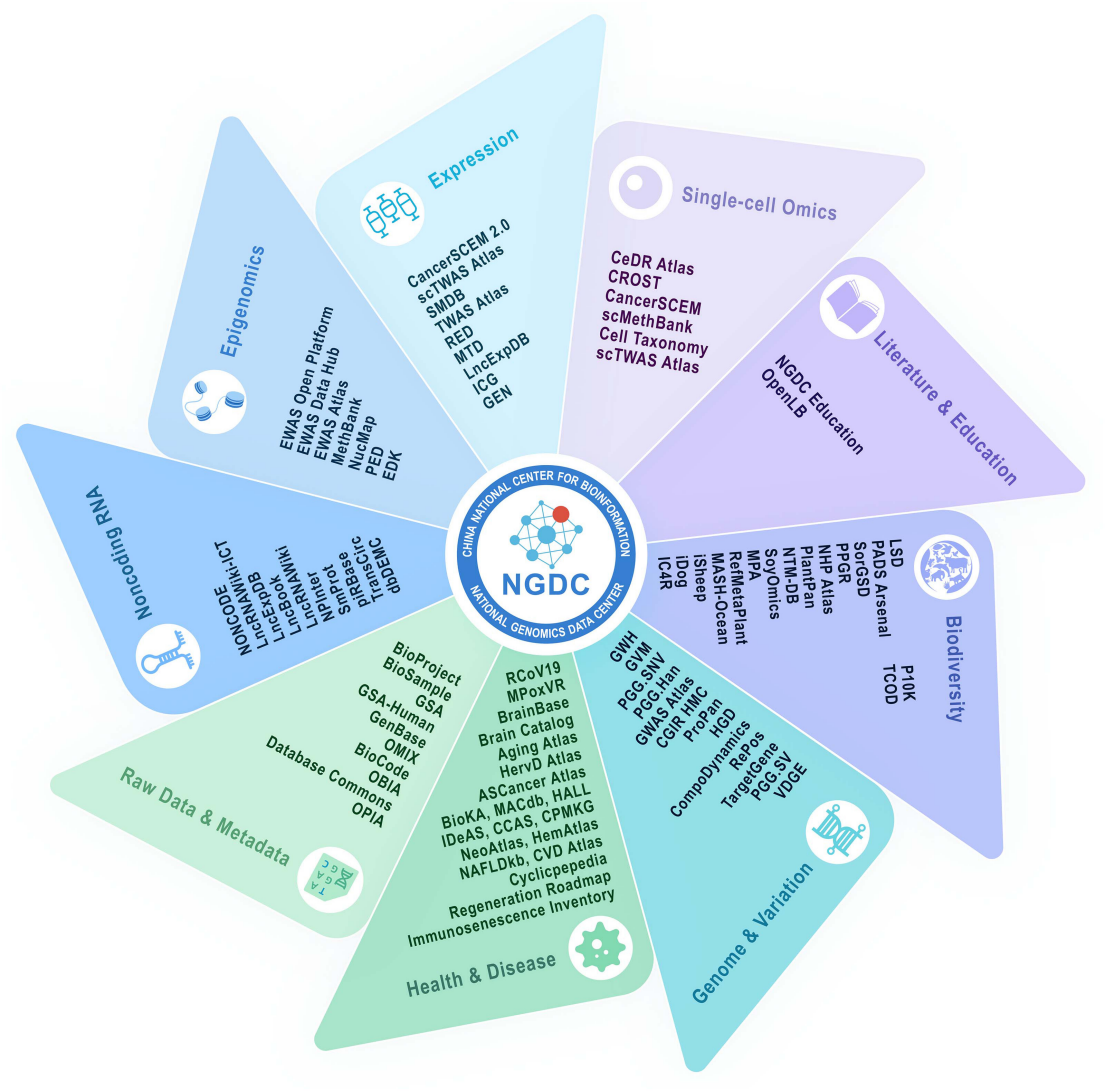
The core database resources of CNCB-NGDC are organized into various categories. These database resources are publicly accessible and searchable through the CNCB-NGDC home page at https://ngdc.cncb.ac.cn. A full list of data resources is shown at https://ngdc.cncb.ac.cn/databases.

**Figure 2.**
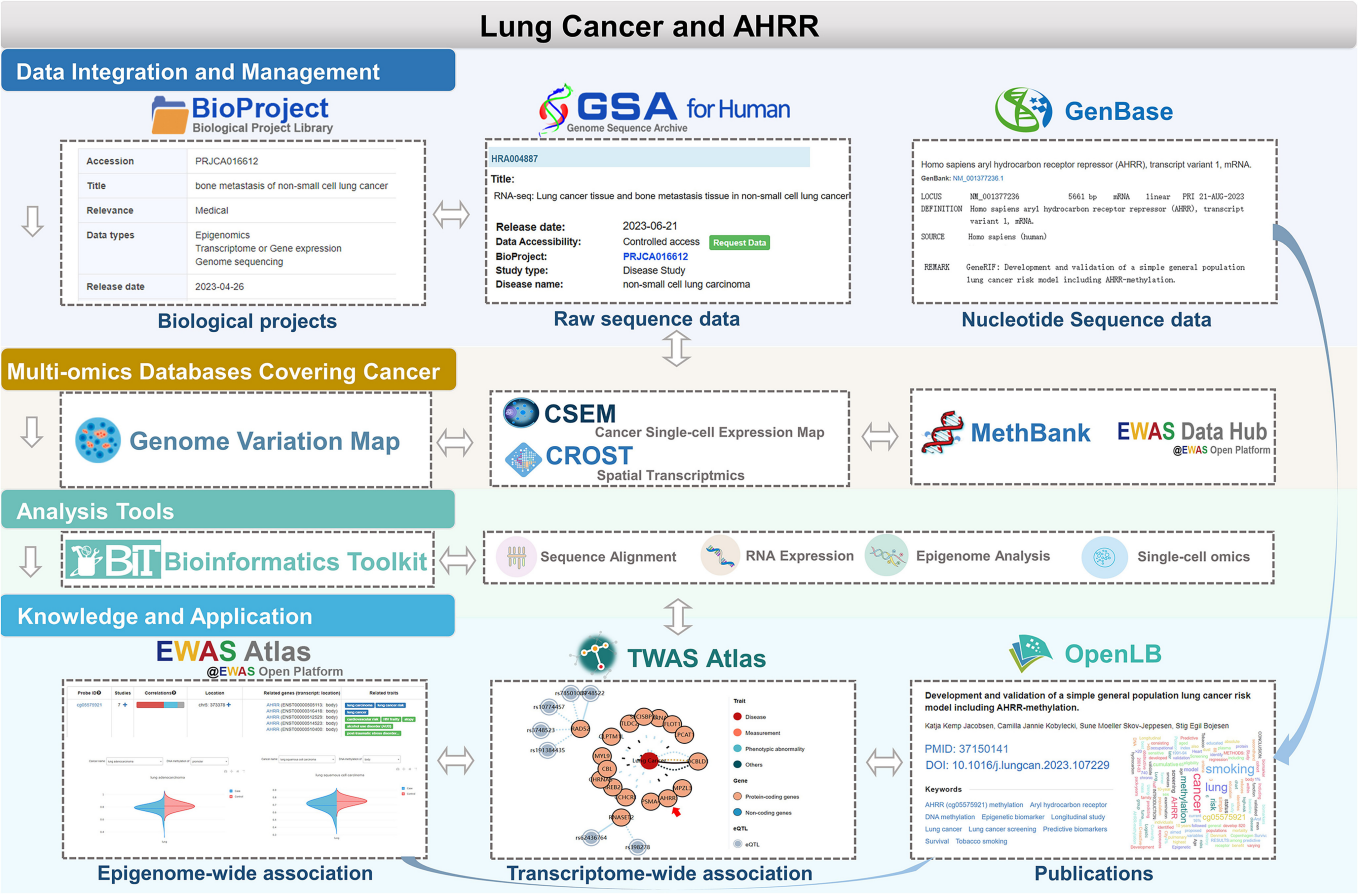
The interconnectivity of CNCB-NGDC’s core databases. The data submission system, multi-omics databases, analytical tools and knowledge repositories are interconnected, allowing users to easily navigate between databases and access relevant information. For instance, the BioProject ID for lung cancer research in multi-omics is PRJCA016612 (https://ngdc.cncb.ac.cn/bioproject/browse/PRJCA016612), which corresponds to the omics data in GSA-human (https://ngdc.cncb.ac.cn/gsa-human/browse/HRA004887). The related gene aryl-hydrocarbon receptor repressor（AHRR）of lung cancer is cross-referenced in the GenBase (https://ngdc.cncb.ac.cn/genbase/search/gb/NM_001377236.1). Leveraging these data, CNCB-NGDC has developed omics databases covering lung cancer, including genome variation database (GVM), as well as databases for single-cell and spatial transcriptomics (GEN, CSEM and CROST), and epigenetics (MethBank and EWAS Open Platform). Users can utilize bioinformatics toolkits like BIT to mine multi-omics data associated with lung cancer. Data analysis and publication curation have further identified changes in AHRR methylation linked to lung cancer (https://ngdc.cncb.ac.cn/ewas/browse?target=traits,https://ngdc.cncb.ac.cn/ewas/datahub/gene/15524), along with a knowledge graph illustrating changes in AHRR expression (https://ngdc.cncb.ac.cn/twas/knowledgegraph). Additionally, literature in OpenLB is associated with the application of the AHRR-based lung cancer risk model (https://ngdc.cncb.ac.cn/openlb/publication/OLB-PM-37150141).

With the increasing volume, scale and complexity of data, the global research community has heightened its demand for the sharing, interoperability and integrated analysis of multi-omics data. Over the past year, CNCB-NGDC has been committed to developing new resources and continuously updating existing resources in aid of advancing global life and health sciences (17–39). The Genome Sequence Archive (GSA), a repository for archiving omics raw data, has been successfully selected in the Global Core Biodata Resource (GCBR) list, initiated by the Global Biodata Coalition (GBC). Additionally, CNCB-NGDC continues to collaborate closely with the International Nucleotide Sequence Database Collaboration (INSDC) for data sharing and exchange. Here, we provide a brief overview of the latest developments and updates at CNCB-NGDC and describe its core resources and services (Figure 1). Notably, these core resources are intricately linked, creating an extensive network that enables users to effortlessly navigate between different databases, access pertinent information and conduct thorough investigations (Figure 2). All resources and services are publicly accessible on the CNCB-NGDC homepage (https://ngdc.cncb.ac.cn).

## New developments

### Single-cell omics

#### scTWAS Atlas

The single-cell transcriptome-wide association studies (scTWAS) Atlas (https://ngdc.cncb.ac.cn/sctwas/) is a comprehensive and specialized database that curates and presents knowledge derived from scTWAS (elsewhere in this issue). The Atlas encompasses 2 765 211 associations spanning 34 traits, 30 cell types, 9 cellular conditions and 16 470 genes, sourced from five publications, ten single-cell eQTL datasets and fifteen GWAS datasets. The scTWAS Atlas platform allows users to construct multi-omics regulatory networks at the cellular level by integrating single-cell expression quantitative trait loci (sc-eQTL) and scTWAS data. Additionally, it provides Manhattan plots for visualizing the distribution and concentration of TWAS gene significance across chromosomal regions. Furthermore, the database enables cross-cell-type analyses to explore cell-type specificity and shared genetic mechanisms of TWAS genes, while incorporating summary data-based Mendelian Randomization analyses to validate gene-trait associations. The scTWAS Atlas serves as a vital resource for investigating genetic mechanisms at single-cell resolution, elucidating the roles of distinct cell types in diverse biological processes and their impact on human health and disease.

### Genome and variation

#### VDGE

The Variation Database of Gene-Edited Animals (VDGE; https://ngdc.cncb.ac.cn/vdge) is a comprehensive, open-access repository that systematically curates and integrates genomic variation and annotation data from a wide range of gene-edited animal species, with a strong emphasis on larger animals that have significant application potential (elsewhere in this issue). VDGE provides extensive genome variation data for each animal trio by utilizing a standardized analysis pipeline based on deep whole-genome sequencing (WGS) data and parent-offspring trio analysis. The database is organized into six key modules: Species, Animal Trios, WGS Samples, On-target Events, Variations and Genes. In its current release, VDGE hosts 115 710 variations and 56 on-target events, meticulously identified from 107 animal trios derived from 175 samples across four species. Additionally, 12 708 genes associated with these variations are annotated in the database. This integrated resource supports advanced phenotype analysis, safety evaluations and translational studies for gene-edited animals.

### Health and disease

#### CVD Atlas

The CVD Atlas (https://ngdc.cncb.ac.cn/cvd) is a comprehensive and curated database consolidating extensive knowledge and data related to cardiovascular diseases (elsewhere in this issue). It integrates information from manual curation, large-scale data analysis and existing databases. The current version comprises 214 731 associations drawn from 309 publications, 652 datasets and 7 databases, encompassing 190 diseases, 44 traits, 36 165 genes, 457 286 SNPs, 8436 differentially methylated positions, 453 differentially expressed proteins and 148 differentially expressed metabolites. The platform also offers an interactive knowledge graph that integrates disease-gene associations and provides two types of analysis tools. Overall, the CVD Atlas is an essential resource that facilitates the use and accessibility of information and knowledge for CVD, benefiting human health and CVD research communities.

#### CPMKG

CPMKG (https://www.biosino.org/cpmkg) is a condition-based knowledge graph designed for precision medicine, offering a valuable resource for clinical research (40). It includes 307 614 meticulously curated knowledge entries across thousands of drugs, diseases, phenotypes, genes and genomic variations, focusing on four key areas: drug side effects, sensitivity, mechanisms and indications. The platform enables drug-centric exploration and multi-knowledge inference, facilitating accelerated knowledge discovery. Key applications include (i) personalized drug recommendations tailored to genetic profiles, side effects and predicted efficacy, (ii) a medication synergy assistant for selecting effective drug combinations with minimized risk and (iii) a pharmacogenomics module providing insights into gene expression, drug–gene interactions and polymorphisms. CPMKG also incorporates a large language model (LLM) that interprets subgraphs, bridging structured data with natural language explanations.

#### Immunosenescence Inventory

Immunosenescence Inventory (https://ngdc.cncb.ac.cn/iaa/) is a multi-omics database for immune aging research (elsewhere in this issue). This comprehensive resource features curated, multidimensional datasets focused on immune senescence. It includes cellular-resolution gene expression profiles for 59 immune cell types across 13 tissues from four species, generated via single-cell RNA sequencing (scRNA-seq), as well as a genome browser with 485 512 epigenomics probes spanning six immune organs, tissues or cells. Additionally, it encompasses bulk RNA sequencing (RNA-seq) data for 54 592 genes across 30 tissues, 22 cell types, 37 immune functions and 2 genders. The Immunosenescence Inventory was built upon the foundation of the Aging Biomarker Consortium (ABC) (28,41). By aggregating diverse and rich datasets from various species across different stages of life, the Immunosenescence Inventory aims to provide a more nuanced and detailed understanding of the aging immune system.

#### HemAtlas

HemAtlas (https://ngdc.cncb.ac.cn/hematlas/) is an interactive multi-omics database for comprehensive mapping of hematopoiesis across developmental stages, species and models. The current version integrates 94 multi-omics datasets from 43 publications, encompassing 1 216 899 cells/samples across 359 major cell types. HemAtlas provides an intuitive visualization platform based on various sequencing technologies, including bulk RNA-seq, scRNA-seq, transposase-accessible chromatin sequencing (ATAC-seq), single-cell ATAC-seq (scATAC-seq), chromatin immunoprecipitation sequencing (ChIP-seq) and spatial transcriptomics. Furthermore, based on the scRNA-seq data, HemAtlas offers organ-wide hematopoietic references through integrative strategy for human, mouse and zebrafish. A series of tools are constructed to elucidate the ontogeny of hematopoiesis across species and offer insights for the generation of hematopoietic stem and progenitor cells（HSPCs) *in vitro*. Additionally, HemAtlas offers a detailed cross-stage developmental map of HSPCs, revealing stage-specific characteristics critical to hematopoiesis. In summary, HemAtlas serves as a comprehensive encyclopedia of hematopoiesis to advance our understanding of hematopoiesis.

#### NeoAtlas

NeoAtlas (https://ngdc.cncb.ac.cn/neoatlas) is a comprehensive database focused on noncanonical neoantigens and their binding predictions with human leukocyte antigen (HLA). The database aggregates knowledge on noncanonical neoantigens and develops predictive models for antigen–HLA interactions, supporting immunotherapy research and cancer vaccine development. NeoAtlas includes 35 574 non-redundant neoantigen–HLA pairs curated from 14 immunopeptidome studies. It features 33 725 RNA neoantigens, 9928 *cis* protein neoantigens and 4889 *trans*protein neoantigens. Additionally, NeoAtlas integrates the NeoBert model into its platform to provide online, real-time analytical tools for predicting the binding affinity of noncanonical antigens. In summary, NeoAtlas serves as a crucial resource, illuminating the noncanonical aspects of neoantigens and contributing to future clinical applications.

#### Cyclicpepedia

CyclicPepedia (https://www.biosino.org/iMAC/Cyclicpepedia/) is a comprehensive and integrated resource designed to support the early stages of cyclic peptide drug development (42). It consolidates data on 8744 known cyclic peptides, including 8614 with sequences and 7032 with structural details. This repository provides detailed information on cyclic peptide sources, classifications, structures, pharmacokinetics, physicochemical properties, patented drug applications and relevant publications. The standardized, curated data offer valuable benchmark datasets for artificial intelligence applications in cyclic peptide research. CyclicPepedia features user-friendly tools for searching and data processing, including a structure-to-sequence converter (Struc2Seq), sequence-to-structure converter (Seq2Struc), peptide property predictor and format transformation utilities. CyclicPepedia facilitates research on cyclic peptide synthesis, structure and biological activity, advancing cyclic peptide therapeutic development.

#### IDeAS

IDeAS (https://www.biosino.org/ideas/) is a comprehensive and interactive database dedicated to the exploration of dysregulated alternative splicing (AS) events in cancer (43). By integrating data from The Cancer Genome Atlas (TCGA) and multiple Chinese tumor RNA-seq datasets, IDeAS encompasses over 215 000 AS events across 33 tumor types. The database includes data from 9913 tumor samples and 730 adjacent normal samples in the TCGA project, along with 923 tumor samples and 556 normal samples from 11 Chinese tumor studies. IDeAS offers an intuitive interface, enabling users to search and visualize cancer-associated AS events while providing tools for survival and clinical indicator analyses. Additionally, the platform incorporates data on splicing factor binding sites and their functional impacts, facilitating the identification of upstream regulators driving cancer-related AS events.

### Biodiversity and biosynthesis

#### RefMetaPlant

RefMetaPlant (https://www.biosino.org/RefMetaDB/) is a comprehensive public database that integrates reference metabolome data for plants and provides advanced metabolite analysis (44). It houses 1 086 068 experimental mass spectra from tissue samples of 153 plant species across five major phyla—Bryophyta, Lycopodiopsida, Pteridophyte, Gymnospermae and Angiospermae—obtained via ultra-performance liquid chromatography-tandem mass spectrometry (UPLC-MS/MS). To standardize data from various plant tissues and organs, RefMetaPlant develops a method for assembling reference metabolomes and built reference datasets for these species. The database also includes 383 759 biologically relevant compounds and 325 103 mass spectral data for standard compounds, of which 135 464 are experimental reference spectra and 189 639 are *in silico* spectra. RefMetaPlant offers a user-friendly web interface featuring tools such as ‘LC-MS/MS Query,’ ‘RefMetaBlast’ and ‘CompoundLibBlast’ for the retrieval and analysis of plant metabolomes and metabolite identification.

#### MASH-Ocean

MASH-Ocean (https://www.biosino.org/mash-ocean/) integrates and analyzes oceanic microbiome and environmental data through the iMAC/iMAC + system, creating the comprehensive Microbiome Atlas/Sino-Hydrosphere for Ocean Ecosystems (45). It offers public access to datasets with unique features tailored to marine microorganisms, including depth-specific selection and comparative analysis between deep-sea and shallow-sea ecosystems, as well as specialized environments such as cold seeps and hydrothermal vents. The project has successfully developed its dataset construction strategy, incorporating over 2000 metagenomic datasets as a foundation, with additional data under processing. Rigorous quality control ensures the reliability of this resource, and large-scale data mining efforts have led to the discovery of new types of photosynthetic microorganisms, significantly advancing our understanding of marine microbial life.

### Tools

#### CCLHunter

CCLHunter (https://ngdc.cncb.ac.cn/cclhunter/) is a data-based authentication platform designed to tackle the complexities of identifying genetically similar or derivative cell lines from the same individual (46). By integrating genetic and expression data, CCLHunter minimizes noise interference and ensures reliable authentication results. It analyzes 1389 human cancer cell lines from CCLE and COSMIC, achieving an overall authentication accuracy of 93.27%. The platform is especially good at authenticating related cell lines, with an accuracy rate of 89.28%. CCLHunter supports high-throughput data processing and provides detailed insights into cell line lineage relationships, all accessible through a user-friendly web server. Overall, CCLHunter enhances the precision of cell line authentication and broadens its applicability and effectiveness in scientific research and drug development.

## Recent updates

### Raw data and metadata

#### BioProject and BioSample

**Figure 3.**
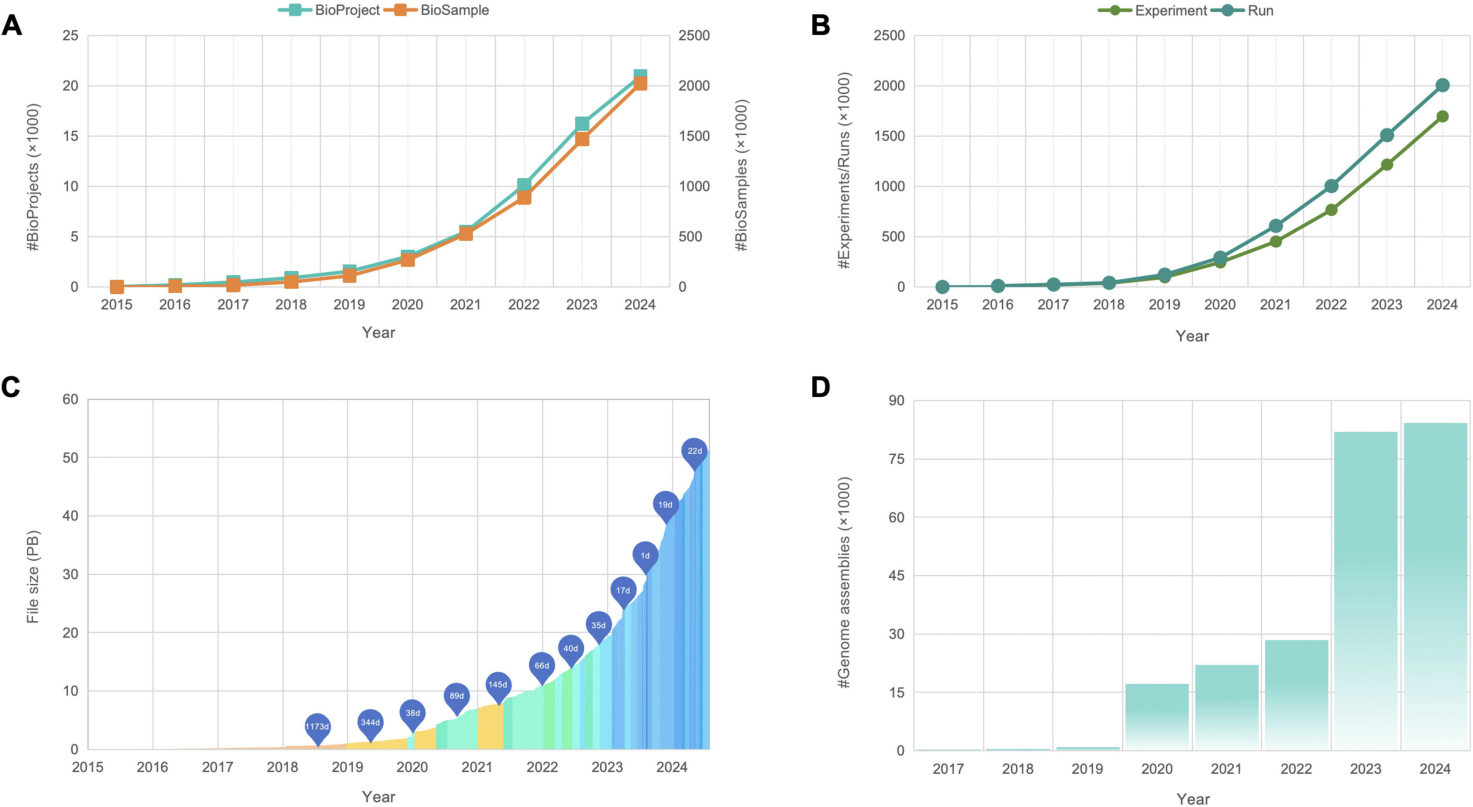
Statistics of data submissions to CNCB-NGDC. (**A**) Data statistics of BioProject and BioSample. (**B**) Data statistics of Experiments and Runs in GSA. (**C**) Timeline of data growth in GSA. (**D**) Statistics of genome assemblies in GWH. All statistics are regularly updated and publicly accessible at https://ngdc.cncb.ac.cn/bioproject, https://ngdc.cncb.ac.cn/biosample and https://ngdc.cncb.ac.cn/gsa and https://ngdc.cncb.ac.cn/gwh.

BioProject (https://ngdc.cncb.ac.cn/bioproject) and BioSample (https://ngdc.cncb.ac.cn/biosample) are centralized public repositories for biological research projects and sample metadata. These platforms provide integrated access to detailed descriptions of biological projects and samples from various experiments, with cross-referenced links to related data resources. As of August 2024, BioProject and BioSample have collected 20 833 projects and 2 001 551 samples from 11 074 users across 2077 organizations (Figure 3), demonstrating significant growth from last year’s 13 487 projects and 1 244 954 samples. Additionally, they have incorporated 775 764 projects and 39 468 828 samples from the INSDC data at NCBI.

#### GSA and GSA-human

The GSA (https://ngdc.cncb.ac.cn/gsa) (47,48) is an open-access repository for non-human raw sequence reads, which provides global communities with free and open services for data submission, data storage and data sharing. GSA for Human (GSA-Human; https://ngdc.cncb.ac.cn/gsa-human) (48,49), a sub-database of GSA, is a data repository dedicated to human genetic omics data with controlled access and security services. As of August 2024, GSA and GSA-Human have collectively accumulated 1 692 749 experiments, 2 002 611 runs and a total of 52.2 PB of data, marking a significant increase from the previous year’s totals of 1 032 023 experiments, 1 232 648 runs and 29.6 PB of data. In addition, GSA has integrated 30 743 097 experiments, 32 680 951 runs and 7.7 PB of raw sequence files from the INSDC’s data resources. To enhance user experience, in 2024, GSA developed a new retrieval system that enables users to conduct complex searches across multiple search fields, filter the search results using a variety of filtering criteria and download the search results with various formats.

#### OMIX

The Open Archive for Miscellaneous Data (OMIX; https://ngdc.cncb.ac.cn/omix) (48), a member of the GSA family, is a versatile and robust data repository specifically designed for the collection, publication and sharing of diverse scientific datasets across the biological research community. Committed to the FAIR (Findable, Accessible, Interoperable and Reusable) principles, OMIX ensures that data are well-structured, easily accessible and reusable across different platforms. As of August 2024, OMIX has significantly expanded its collection to 5224 datasets, encompassing 26 936 individual files and surpassing 82.48 TB of data, a substantial growth from last year’s 3384 submissions, 15 837 files and 59.34 TB of data.

#### GenBase

GenBase (https://ngdc.cncb.ac.cn/genbase) is a user-friendly portal for archiving, searching and sharing of nucleotide and protein sequences (25). It ensures data integrity and enhances data reusability through rule-based automatic quality control and expert-based manual curation, mostly compatible with INSDC standards for submitted data (2). As of August 2024, GenBase has processed 81 929 nucleotide sequences and 832 740 annotated protein sequences, showing significant growth from last year’s 37 981 nucleotides and 362 296 protein sequences, submitted by 309 researchers from 197 institutions. Of these, 76 340 nucleotide sequences (93%) and 723 863 protein sequences (87%) have been publicly released. Particularly, GenBase has received and released 60 578 severe acute respiratory syndrome coronavirus 2（SARS-CoV2） genome sequences with standard standardized annotations. Additionally, it integrates over 580 million nucleotide and protein sequences from INSDC, facilitating efficient data access for domestic researchers. The latest version includes an online update feature for released sequences, ensuring data accuracy and enhancing user experience.

#### Database Commons

Database Commons (https://ngdc.cncb.ac.cn/databasecommons) is a categorized catalog of worldwide biological databases, providing impact assessment and valuable statistics (50). Currently, it catalogs 6918 biological databases, linking to 10 399 publications across 2309 organizations. This represents growth from August 2023, which included 6354 databases and 9808 publications. To account for differences in database age, Database Commons introduces the z-index, representing the average annual citation rate. Based on the z-index, DAVID, KEGG, cBioPortal, STRING, AlphaFold DB and gnomAD emerge as top performers, highlighting research focus on human genome studies in precision medicine and AI applications in life sciences. Additionally, Database Commons has introduced a browsing feature that sorts database entries by manual update time or creation time, helping users access the latest updates and newly added databases.

### Genome and variation

#### Genome Warehouse

The Genome Warehouse (GWH; https://ngdc.cncb.ac.cn/gwh) serves as an essential public repository, archiving a wealth of genome assembly sequences, annotations and associated metadata (51). As of August 12, 2024, GWH has accepted 84 262 genome assemblies from animals, plants, fungi, protists, bacteria, archaea and metagenomes. Among them, 56 885 assemblies (up from 19 270 last year) from 3326 organisms were released and published in 426 articles (up from 278) across 107 scientific journals, submitted by 1022 providers from 360 organizations in 7 countries/regions. To enhance user experience, this version of GWH introduces an online batch submission feature and an updated quality control system for rigorous genomic content review. This update of GWH also incorporates an automated reannotation pipeline leveraging the Prokaryotic Genome Annotation Pipeline (PGAP) (52) from NCBI (53) to deliver standardized genome reannotations. These updates improve the efficiency and reliability of data submission and retrieval, adding significant value to genomics research.

#### GVM

The Genome Variation Map (GVM; https://ngdc.cncb.ac.cn/gvm) serves as a repository for genome variations, including single-nucleotide polymorphisms (SNPs) and small insertions and deletions (INDELs) (54,55). It is featured by the collection and submission of genomic variation data from a wide-range species around the world. As of September 2024, GVM has archived ∼1.6 billion variants from 57 species, encompassing 391 projects and 83 366 samples, which were manually curated and analyzed in a standardized pipeline. GVM has also received 623 data submissions covering 471 128 samples from 175 organizations. A significant enhancement in GVM is the addition of a data request management system to facilitate communication between data owners and applicants for controlled access data. Moreover, a new haplotype phasing tool for real-time online data analysis has been introduced, further enabling researchers to fully utilize GVM data.

#### GWAS Atlas

GWAS Atlas (https://ngdc.cncb.ac.cn/gwas) focuses on variants knowledge of genome-wide variant–trait association (GWAS) (56,57). It has integrated 302 295 associations across 26 cultivated plants and 5 domesticated animals that were manually curated from 3828 studies in 922 publications. Compared to its previous version, GWAS Atlas has 50% more species, and newly incorporates 24 186 associations, which relate to 706 different traits and 18 445 variations. Additionally, GWAS Atlas newly launched a data submission feature at the beginning of 2023. Till now, it has archived 32 GWAS project submissions from 17 organizations. Together, GVM and GWAS Atlas have been improving their data volume and functionality, and both are valuable resources for genomic variation research of important traits.

### Health and disease

#### RCoV19

The 2019 Novel Coronavirus Resource (RCoV19; https://ngdc.cncb.ac.cn/ncov) (58–61) serves as an advanced platform for integrating, tracking mutations and issuing pre-alerts for high-risk variants of SARS-CoV-2. As of August 2024, RCoV19 has integrated over 17.6 million de-duplicated SARS-CoV-2 genome sequences along with their metadata, identifying 7.7 million complete and high-quality genomic sequences. Leveraging this extensive dataset, RCoV19 amalgamates mutation effect analysis with the temporal dynamics of haplotype evolutionary networks, utilizing machine learning algorithms to issue weekly alerts for potential high-risk variants. In the initial half of 2024, the platform adeptly completed 27 early warning analyses, accurately predicting high-risk variants, including JN.1 and KP.3.1, thereby securing a crucial timeframe for epidemic prevention and control. Furthermore, through the profound integration of manual curation and bioinformatics technology, RCoV19 systematically analyzes six pivotal areas: transmissibility, antibody escape, drug sensitivity, pathogenicity, structural stability and T-cell epitope variation. To date, it has accumulated a total of 12 554 detailed entries on mutation effect, greatly enhancing the comprehension of SARS-CoV-2 mutation mechanisms and offering an indispensable reference framework for scientific research and prevention strategy formulation.

#### MPoxVR

The Monkeypox Virus Resource (MPoxVR; https://ngdc.cncb.ac.cn/gwh/poxvirus) (62) is a one-stop platform for Monkeypox virus sequence integration and sequence variants identification and annotation. The platform features an automatic pipeline for sequence integration and variation analyses, enabling daily data updates since its launch. As of August 2024, MPoxVR has collected detailed information on over 7700 Monkeypox virus genome sequences and nearly 60 000 genomic variations, all of which are browsable, searchable and downloadable from the website. This year, we have incorporated an enhanced feature that allows for the identification of common variants in Monkeypox virus sequences, along with dynamic analyses of the temporal and county-level distribution of these genomic variants. Altogether, MPoxVR will function as a valuable resource for relevant studies and epidemic constraints.

### Expression

#### CancerSCEM

The Cancer Single-cell Expression Map (CancerSCEM; https://ngdc.cncb.ac.cn/cancerscem) is a public database that integrates, analyzes and visualizes scRNA-seq data of human pan-cancers (63). As of August 2024, the database hosts 1466 scRNA-seq datasets from 127 research projects spanning 74 cancer types, showing a significant increase in data compared to the previous version. The database originally included normal samples and samples from healthy peripheral blood as controls for tumor-normal comparative analysis. Additionally, the data analysis has been enhanced with four new transcriptome-level analyses and a range of up-to-date metabolic profiling, including copy number variation (CNV) evaluation, transcription factor (TF) enrichment, pseudotime trajectory construction, diverse biological features scoring, metabolic flux inference, metabolic dynamic variance tracking and metabolic correlation measurement, which deepen our understanding of complex tumor biology at single-cell resolution. Furthermore, the functionality of CancerSCEM has been expanded with a metabolic-dedicated page for visualizing results and an interactive analysis platform with 4 modules and 14 functions. These comprehensive updates position CancerSCEM as an indispensable database for tumor scRNA-seq data utilization and to further support clinical practice.

### Epigenomics

#### EWAS Open Platform

EWAS Open Platform (https://ngdc.cncb.ac.cn/ewas) is a continuously evolving resource for epigenome-wide association study (EWAS) that combines data, knowledge and a toolkit (64). The latest update introduces a new causal relationship module based on MR analysis to better identify true causal links in epigenetic associations crucial for disease onset and progression. This module encompasses 12 402 causal relationships involving DNA methylation, gene expression, traits and diseases, covering conditions like Alzheimer’s disease, type 2 diabetes, heart disease and various cancers. The platform has also added 13 235 DNA methylation microarray data that have undergone batch effect correction (65,66) and updated its knowledge repository with 100 446 high-quality epigenetic associations (67). Overall, the EWAS Open Platform now integrates 752 193 epigenetic associations related to 832 traits from 1121 publications and supports combined searches and downloads of 159 944 methylation data. These updates improve the understanding of epigenetics in disease and support research into underlying mechanisms and potential therapies.

#### MethBank

The Methylation Bank (MethBank; https://ngdc.cncb.ac.cn/methbank) (68–70) is a comprehensive database of DNA methylation across multiple species and diverse biological contexts. Since its last release in September 2023, MethBank has expanded its data by 69%, now including an additional 435 animal samples (from *Homo sapiens, Bos taurus* and *Ovis aries*) and 1015 plant samples (from *Fragaria vesca, Arabidopsis thaliana* and *Glycine max*). The database integrates whole-genome single-base resolution methylomes from 3552 high-quality samples across 26 species. The latest update introduces a new cancer module that documents differentially methylated regions (DMRs) from 12 common cancer types, including prostate, breast and colon cancer. Annotations for these DMRs now include resources such as enhancers, silencers and transcription factors. MethBank also features a total of 604 methylation tools, including 71 new additions. These updates advance research into DNA methylation’s roles in disease, development and environmental contexts.

### Non-coding RNA

#### LncBook and circAtlas

LncBook (http://ngdc.big.ac.cn/lncbook) features providing a comprehensive list of human long non-coding RNAs （lncRNAs） with extensive annotations at multiple omics levels (71). Since the release of version 2.0, LncBook has made significant advancements by integrating newly identified lncRNAs from 10 expert databases and by identifying full-length lncRNA transcripts using 94 PacBio long-read RNA sequencing datasets. This effort has resulted in a significant increase in the number of lncRNAs, rising to 526 318 from 323 950 in version 2.0. Among these, there are 148 353 full-length lncRNAs supported by long-read assembly, including 69 517 that are validated, 4496 with corrected boundaries and 74 340 novel assemblies. This information is detailed in the version 2.1 GTF file.

The latest version of the circAtlas database (https://ngdc.cncb.ac.cn/circatlas/) now includes over 3.1 million circular RNA（circRNAs） from a comprehensive compendium of 2609 Illumina and 65 nanopore RNA-seq datasets from 33 diverse tissues within 10 distinct species. circAtlas 3.0 (72) addresses the existing gap by offering the most extensive collection of circRNAs, along with their expression and functional profiles in vertebrates. This provides a solid foundation for circRNA research and serves as an excellent starting point for exploring their biological significance.

#### LncExpDB

LncExpDB (https://ngdc.cncb.ac.cn/lncexpdb) integrates and rigorously curates expression profiles of human lncRNAs across a wide range of biological contexts (73). Utilizing the lncRNA gene reference from LncBook (71), LncExpDB evaluates the expression reliability and potential of lncRNA genes, identifying featured genes across nine biological contexts. This year’s update introduces three additional biological contexts—immunotherapy, aging and metabolic diseases—along with 24 related biological conditions, leading to the addition of 1374 featured genes and 3262 highly expressed lncRNA genes. Enhanced visualization of expression profiles is now available for these new contexts. Additionally, we have incorporated a ‘Pipeline’ module to share commands and parameters used for lncRNA expression profiling analysis for users’ reference.

#### LncRNAWiki-ICT

To streamline manual editing for efficient and rapid lncRNA literature curation in LncRNAWiki (https://ngdc.cncb.ac.cn/lncrnawiki/) (74), the intelligent tool LncBot has been developed. It employs a state-of-the-art open-source LLM and vector embedding model, utilizing retrieval-augmented generation (RAG) to extract functional information from lncRNA literature based on the existing curation model of LncRNAWiki. Additionally, it traces and maps the information extracted by the LLM to the corresponding locations in the PDF files, facilitating verification by curators. In summary, LncBot automates the curation workflow, significantly reducing the burden on curators.

### Biodiversity

#### SoyOmics

SoyOmics (https://ngdc.cncb.ac.cn/soyomics) is an integrated multi-omics database for soybeans designed to provide a one-stop solution for big data mining (38). Compared with the version in 2023, in-depth updates have been conducted on its transcriptome module. First, new gene expression data for 314 samples from ZH13 have been launched, covering 13 tissues across 13 different developing stages, which give a detailed landscape of the soybean transcriptome profiles and facilitate a comprehensive understanding of soybean development. Second, five spatially enhanced REsolution omics sequencing (Stereo-seq) datasets from various tissues have been newly released. Third, seven single-nucleus RNA sequencing (snRNA-seq) datasets for five tissues have been newly implemented. These datasets capture the spatial information of gene expression patterns and offer a deeper insight into tissue architecture, cell-to-cell communication and cell heterogeneity.

#### iDog

iDog (https://ngdc.cncb.ac.cn/idog/) is a comprehensive public resource for domestic dogs (*Canis lupus familiaris*) and wild canids (75). It aims to collect and integrate multi-omics data, providing a variety of data services to the global canine research community. The current version of iDog houses approximately 29.55 million SNPs and 16.54 million INDELs from 1929 modern samples. In addition, it newly incorporates 29.09 million SNPs from 111 ancient canis DNA, 43 487 breed-specific SNPs and 530 disease/trait-associated variants. Moreover, 141 BioProjects related to gene expression have been newly analyzed. Meanwhile, iDog includes a new single-cell transcriptome module with 105 057 cells from the dog hippocampus, a new DNA methylation module that evaluates methylation levels across 547 samples, and a new chromatin accessibility module with peak information for 87 samples. Moreover, phenotype information for 897 dog diseases, 3207 genotype-to-phenotypes pairs and 349 dog disease-associated genes have been curated, supplemented by two ontologies constructed for standardizing breed and disease Additionally, 13 new tools have been appended for various analyses. Its well-structured data organization, user-friendly interfaces and various online tools make it an indispensable resource for researchers, dog owners and veterinarians within the dog community.

### Tools

#### OpenLB

The Open Library of Bioscience (OpenLB; https://ngdc.cncb.ac.cn/openlb) offers users convenient and open access to a vast array of biological literature. The current version features over 37 million accessible abstracts from resources like PubMed (76) (https://pubmed.ncbi.nlm.nih.gov/), bioRxiv (https://www.biorxiv.org/) and medRxiv (https://www.medrxiv.org/). OpenLB supports rapid full-text and advanced search capabilities, allowing users to apply customizable search conditions for efficient publication retrieval. Additionally, it provides related data information and links to CNCB-NGDC resources, along with functionalities such as similar literature recommendations, keyword cloud generation for abstract, citation tracking via the Dimension API (https://dimensions.ai) and entity recognition through PubTator 3.0 (77), delivering a comprehensive and diverse set of practical functions to enhance the user experience.

## Concluding remarks

This year, CNCB-NGDC has achieved a significant milestone with its GSA being successfully included in the GCBR list and the establishment of three specialized subcenters for data consolidation and aggregation. This achievement underscores CNCB-NGDC’s ongoing commitment to advancing the life sciences by providing a comprehensive suite of innovative and continuously updated database resources. These meticulously developed resources, particularly the databases closely related to human health and disease, aim to facilitate broad sharing, integration and application of multi-omics data, encompassing data archiving, curation, and analysis and driving transformative advancements in life, health and medicine sciences, particularly in precision medicine, and beyond.

Looking ahead, CNCB-NGDC will further enhance its resources and services by automating data submission workflows, improving data management and integration capabilities, upgrading infrastructure for efficient big data storage and transmission, and developing new tools and pipelines for in-depth multi-omics data analysis. Through its robust data infrastructure and unwavering commitment to scientific excellence, CNCB-NGDC provides fundamental support in aiding worldwide researchers to uncover new insights and discoveries for personalized medicine, precise diagnostics, drug development, plant breeding and biosafety.

## Data Availability

All resources and services are publicly available on the home page of CNCB-NGDC (https://ngdc.cncb.ac.cn).

## Appendix

**Corresponding author:** Yiming Bao^1,2,3,*^

**Co-corresponding authors:** Zhang Zhang^1,2,3,*^, Wenming Zhao^1,2,3,*^, Jingfa Xiao^1,2,3,*^, Shuhui Song^1,2,3,*^,Shunmin He^4,*^, Guoqing Zhang^5,3,*^, Yixue Li^5,6,*^, Guoping Zhao^5,7,*^, Runsheng Chen^4,*^

**CNCB-NGDC MEMBERS**(Arranged by project role and then by contribution except for Team Leader (TL), as indicated)

**scTWAS Atlas:** Jialin Mai^1,2,3,#^, Qiheng Qian^1,2,3,#^, Hao Gao^1,2,3,#^, Zhuojing Fan^1,2^, Jingyao Zeng^1,2,#^, Jingfa Xiao^1,2,3,*^ (TL)

**VDGE:** Wenwen Shi^8,#^, Enhui Jin^1,2,3,#^, Lu Fang^9, #^, Yanling Sun^1,2,10,11^, Zhuojing Fan^1,2^, Junwei Zhu^1,2^, Chengzhi Liang^3,9^, Yaping Zhang^12^ , Yongqing Zhang^8,3,13,#^, Guodong Wang^3,12,#^, Wenming Zhao^1,2,3,*^

**CVD Atlas:** Qiheng Qian^1,2,3,#^, Ruikun Xue^1,2,3,#^, Chenle Xu^1,2,3^, Fengyu Wang^14^, Jingyao Zeng^1,2^, Jingfa Xiao^1,2,3,*^ (TL)

**CPMKG:** Jiaxin Yang^5,3,#^, Xinhao Zhuang^5,3,#^, Ping Xu^5,3,#^, Yunchao Ling^5,#^, Guoqing Zhang^5,3,*^

**Immunosenescence Inventory:** Hao Li^15,2,3,#^, Wei Zhao^1,2,3,#^, Fei Yang^1,2,#^, Qin Qiao^15,2,#^, Shuai Ma^16,3,17,18,#^, Kuan Yang^15,3,19,#^, Si Wang^20,21,18,22^, Jing Qu^16,23,3,24,17,18,#^, Guanghui Liu^16, 3,24,17,20,21,18,#^, Yiming Bao^1,2,3,^*, Weiqi Zhang^15,2,3,19,17,18,#^

**HemAtlas:** Zhixin Kang^17,25,3,#^, Tongtong Zhu^1,2,3,#^, Dong Zou^1,2,#^, Yifan Zhang^17,25,3^, Mengyao Liu^26^, Suwei Gao^17,25,3^, Xiaohan Wang^17,25,3^, Shuai Jiang^1,2^, Lu Wang^26^, Zhang Zhang^1,2,3,*^, Feng Liu^17,25,3,#^

**NeoAtlas:**Fengxian Han^27,28,#^, Haobin Chen^29,#^, Wei Zhao^1,2,3,#^, Meilong Shi^30,#^, Qiaoshuang Chen^31^, Yizhuo Li^32^, Shan Zhang^33^, Lingyun Xu^27,28^, Fei Yang^1,2^, Yiming Bao^1,2,3,*^, Chunman Zuo^29,#^, Jing Li^27,28,31,34,#^

**CyclicPepedia:** Lei Liu^35,#^, Liu Yang^36,#^, Guoqing Zhang^5,3,*^, Ruixin Zhu^35,#^, Dingfeng Wu^36,#^

**IDeAS:** Hanwen Zhou^5,3,#^, Liyun Yuan^5,#^, Zefeng Wang^5,3,37,38,#^, Guoqing Zhang^5,3,*^

**RefMetaPlant:** Han Shi^3,39,#^, Xueting Wu^39,#^, Yan Zhu^39,#^, Tao Jiang^39,#^, Guoqing Zhang^5,3^, Ping Chen^39,#^, Xuan Li^3,39,#^

**MASH-Ocean:** Yinzhao Wang^40,#^, Liuyang Li^40,#^, Qiang Li^1,2,#^,Guoping Zhao^5,3,*^, Fengping Wang^40,41,#^, Guoqing Zhang^5,3,*^

**CCLHunter:** Congfan Bu^1,2,#^, Xinchang Zheng^1,2,#^, Jialin Mai^1,2,3^, Zhi Nie^1,2,3^, Jingyao Zeng^1,2^, Qiheng Qian^1,2,3^, Tianyi Xu^1,2^, Yanling Sun^1,2^, Yiming Bao^1,2,3,*^, Jingfa Xiao^1,2,3,*^

**BioProject & BioSample & GSA & GSA-Human:** Xu Chen^1,2,#^, Tingting Chen^1,2,#^, Xiaolong Zhang^1,2,#^, Junwei Zhu^1,2^, Lili Dong^1,2^, Yanling Sun^1,2^, Caixia Yu^1,2^, Yubo Zhou^1,2^, Sisi Zhang^1,2^, Zhuojing Fan^1,2^, Shuang Zhai^1,2^, Yubin Sun^1,2^, Qiancheng Chen^1,2^, Xiaoyu Yang^1,2^, Xin Zhang^1,2^, Zhengqi Sang^1,2^, Yonggang Wang^1,2^, Yilin Zhao^1,2^, Huanxin Chen^1,2^, Yanqing Wang^1,2,#^ (TL), Wenming Zhao^1,2,3,*^ (TL)

**OMIX:** Anke Wang^1,2,#^, Caixia Yu^1,2,#^, Yanqing Wang^1,2^, Sisi Zhang^1,2,#^ (TL)

**GenBase:** Congfan Bu^1,2,#^, Xuetong Zhao^1,2,#^, Xue Bai^1,2,#^, Jingfa Xiao^1,2,3^, Zhang Zhang^1,2,3^, Wenming Zhao^1,2,3^, Bixia Tang^1,2^ (TL), Yiming Bao^1,2,3,*^

**Database Commons:** Miaomiao Wang^1,2,3,#^, Shiting Wang^1,2,3,#^, Wenzhuo Cheng^1,2,3,#^, Zheng Luo^1,2,3^, Shaosen Zhang^1,2,3^, Haochen Liu^1,2,3^, Lin Liu^1,2^, Lina Ma^1,2,3,#^ (TL)

**Genome Warehouse:** Xuetong Zhao^1,2,#^, Yingke Ma^1,2,#^, Zhenxian Han^1,2,#^, Meili Chen^1,2,#^ (TL)

**GVM:** Dongmei Tian^1,2,#^, Xue Bai^1,2,#^, Yi Wang^1,2,3,#^, Bixia Tang^1,2,#^, Zishan Wu^1,2,3^, Shuhui Song^1,2,3,*^ (TL)

**GWAS Atlas:** Dongmei Tian^1,2,#^, Xue Bai^1,2,#^, Zishan Wu^1,2,3,#^, Yi Wang^1,2,3^, Shuhui Song^1,2,3,*^ (TL)

**RCoV19:** Cuiping Li^1,2,#^, Lina Ma^1,2,#^, Dong Zou^1,2,#^, Wei Zhao^1,2,3,#^, Xue Bai^1,2,#^, Lun Li^1,2,#^, Junwei Zhu^1,2^, Enhui Jin^1,2,3^, Hailong Kan^1,2,3^, Zhang Zhang^1,2,3^, Wenming Zhao^1,2,3^, Yiming Bao^1,2,3,*^ (TL), Shuhui Song^1,2,3,*^ (TL)

**MPoxVR:** Cuiping Li^1,2,#^, Yingke Ma^1,2,#^, Meili Chen^1,2,#^, Yiming Bao^1,2,3,*^, Shuhui Song^1,2,3,*^ (TL)

**CancerSCEM:** Jingyao Zeng^1,2,#^ (TL), Zhi Nie^1,2,3,#^, Yunfei Shang^1,2,3,#^, Jialin Mai^1,2,3,#^, Yadong Zhang^1,2^, Yuntian Yang^42^, Chenle Xu^1,2,3^, Jing Zhao^1,2,3^, Zhuojing Fan^1,2^, Jingfa Xiao^1,2,3,*^

**EWAS Open Platform:**Fei Yang^1,2,#^, Yiran Zhang^1,2,3,#^, Bing Pei^1,2,3,#^, Zhuang Xiong^43,#^, Shuxian Jiang^44^, Song Wu^1,2,3^,Yaoke Wei^1,2,3^, Haochen Liu^1,2,3^, Huijing Jiang^1,2,3^,Wenting Zong^1,2,3^, Rujiao Li^1,2,3,#^ (TL)

**MethBank:** Mochen Zhang^1,2,#^, Fei Yang^1,2,#^, Dong Zou^1,2,#^, Shuxian Jiang^44^, Rujiao Li^1,2,3,#^ (TL)

**LncBook:** Xinyu Zhou^1,2,3,19,#^, Zhao Li^1,2,3^, Lin Liu^1,2^, Lina Ma^1,2,3,#^ (TL)

**LncExpDB:** Yue Qi^1,2,3#^, Zhao Li^1,2,3,#^, Lina Ma^1,2,3,#^ (TL)

**LncRNAWiki:** Xing Zheng^1,2,3,#^, Lin Liu^1,2^, Zhao Li^1,2,3^, Lina Ma^1,2,3,#^ (TL)

**SoyOmics:** Yanting Shen^45,#^ ,Yucheng Liu^45,#^ , Dongmei Tian^1,2,#^ ,Yang Zhang^1,2,3,#^, Shuhui Song^1,2,3,*^ , Zhixi Tian^3,45,#^

**IDog:**Yibo Wang^1,2,3,#,^ Jiani Sun^1,2,3,19,#^, Demian Kong^1,2,3,#^, Bowen Zhou^12,46,47,#^, Mengting Ding^12,46,47^ ,Yuyan Meng^1,2,3,19^, Guangya Duan^1,2,3^, Ying Cui^1,2,3^, Zhuojing Fan^1,2^, Yaping Zhang^12,46,47^, Yanhu Liu^12,46,#^, Wenming Zhao^1,2,3,*^ and Bixia Tang^1,2,#^ (TL)

**OpenLB:**Dong Zou^1,2^ (TL)

**Writing Group:** Fei Yang^1,2,#^,Shuai Jiang^1,2,#^, Zhuojing Fan^1,2^, Shuhui Song^1,2,3,*^, Wenming Zhao^1,2,3,*^, Jingfa Xiao^1,2,3,*^ , Zhang Zhang^1,2,3,*^, Yiming Bao^1,2,3,*^

**CNCB-NGDC SubCenters** (Listed in alphabetical order by database names)

**NGDC-BDV:**Xuemei Lu^12,48,3^, Yanan Wang^48,3^

**NGDC-TCM:**Yuan Yuan^49,50^, Wei Liu^49^

**NGDC-TGD:**Jinyan Huang^51^

**CNCB-NGDC PARTNERS** (Listed in alphabetical order by database names)

**Animal-APA:** Weiwei Jin^52^, Jing Gong^52^

**Animal-eRNA:** Weiwei Jin^52^, Jing Gong^52^

**Animal-SNPAtlas:** Xiaohui Niu^52^, Jing Gong^52^

**AnimalTFDB:** Wenkang Shen^53^, Anyuan Guo^53^

**BBCancer:** Zhixiang Zuo^54^, Jian Ren^54^

**CancerSEA:** Yun Xiao^55^, Xia Li^55^

**CellMarker:** Yun Xiao^55^, Xia Li^55^

**CGDB:** Dan Liu^56^, Yu Xue^56^

**CGGA**: Zheng Zhao^57^, Tao Jiang^57^

**circAtlas:** Fangqing Zhao^3,58,59^, Jinyang Zhang^58^

**CirFunBase**: Xianwen Meng^60^, Ming Chen^60^

**ConsRM:** Bowen Song^61^, Jia Meng^62^

**CPLM:** Yujie Gou^56^, Miaomiao Chen^56^

**dbPSP & THANATOS:** Di Peng^56^, Yu Xue^56^

**DEG & DoriC:** Hao Luo^63-65^, Feng Gao^63-65^

**DirectRMDB:** Jie Jiang^61,62^, Kunqi Chen^66,67^

**DrLLPS:** Xinhe Huang^56^, Yu Xue^56^

**eLMSG:** Wan Liu^5^, Guoqing Zhang^5,3^

**EPSD:** Chi Zhang^56^, Yu Xue^56^

**EVAtlas:** Chunjie Liu^53^, Anyuan Guo^53^

**EVmiRNA:** Gui-Yan Xie^53^, Anyuan Guo^53^

**GenTree:** Hao Yuan^3,68^, Yong E. Zhang^3,68^

**GTDB:** Chenfen Zhou^5^, Guoqing Zhang^5,3^

**HCL**: Ming Chen^60^, Guoji Guo^69^

**hTFtarget:** Qiong Zhang^53^, Anyuan Guo^53^

**iEKPD:** Shanshan Fu^56^, Miaoying Zhao^56^

**IMP:** Tong Chen^70^, Yuan Yuan^50^

**iPCD:** Dachao Tang^56^, Yu Xue^56^

**iUUCD:** Ming Lei^56^, Yu Xue^56^

**LeukemiaDB:** Mei Luo^53^, Anyuan Guo^53^

**lnCAR:** Yubin Xie^54^, Jian Ren^54^

**lncRNASNP2:** Yaru Miao^53^, Anyuan Guo^53^

**lncRNASNP3**: Anyuan Guo^53^, Jing Gong^52^

**m5C-Atlas:** Jiongming Ma^66^, Kunqi Chen^66^

**m6A-Atlas:** Haokai Ye^61,62^, Kunqi Chen^66^

**m6A-TSHub:** Bowen Song^71^, Daiyun Huang^62^

**m7GHub:** Yuxin Zhang^62,71^, Bowen Song^71^

**MCA**: Ming Chen^60^, Guoji Guo^69^

**MiCroKiTS:** Di Zhang^56^, Jianzhen Peng^56^

**miRNASNP:** Chunjie Liu^53^, Anyuan Guo^53^

**msRepDB:** Xin Gao^72^, Jianxin Wang^73^

**ncRNA-eQTL:** Jiang Li^52^, Jing Gong^52^

**Pancan-mnvQTL:** Xiaohui Niu^52^, Jing Gong^52^

**PEA:** Guiyan Xie^53^, Anyuan Guo^53^

**PceRBase:** Chunhui Yuan^60^, Ming Chen^60^

**PlantRegMap:** Dechang Yang^74^, Ge Gao^74^

**Plant-ImputeDB:** Xiaohui Niu^52^, Jing Gong^52^

**PncStres:** Wenyi Wu^60^, Ming Chen^60^

**PTMD:** Cheng Han^56^, Yu Xue^56^

**RhesusBase:** Juntian Qi^75^, Chuanyun Li^75^

**RMDisease:** Xuan Wang^62^, Zhen Wei^62^**^, 76^**

**RMVar:** XiaoTong Luo^54^, Jian Ren^54^

**ScRAPdb:** Jiaxing Yue^77^, Zepu Miao^77^

**SEECancer:** Yun Xiao^55^, Xia Li^55^

**SEGreg:** Qing Tang^53^, Anyuan Guo^53^

**SNP2APA:** Anyuan Guo^53^, Jing Gong^52^

**THANATOS:** Zihao Feng^56^, Yu Xue^56^

**VFDB:** Bo Liu^78^, Jian Yang^78^

**WERAM:** Chenyu Yang^56^, Leming Xiao^56^

**ZCURVE_CoVdb:** Hao Luo^63-65^, Feng Gao^63-65^

1. National Genomics Data Center, China National Center for Bioinformation, Beijing 100101, China

2. Beijing Institute of Genomics, Chinese Academy of Sciences, Beijing 100101, China

3. University of Chinese Academy of Sciences, Beijing 100049, China

4. Key Laboratory of Epigenetic Regulation and Intervention, Institute of Biophysics, Chinese Academy of Sciences, Beijing 100101, China

5. National Genomics Data Center and Bio-Med Big Data Center, CAS Key Laboratory of Computational Biology, Shanghai Institute of Nutrition and Health, Chinese Academy of Science, Shanghai 200031, China

6. Guangzhou Laboratory, Guangzhou 510005, China

7. Hangzhou Institute for Advanced Study, University of Chinese Academy of Sciences, Hangzhou 310024, China

8. State Key Laboratory of Molecular and Developmental Biology, Institute of Genetics and Developmental Biology, Chinese Academy of Sciences, Beijing 100101, China

9. Key Laboratory of Seed Innovation, Institute of Genetics and Developmental Biology, Chinese Academy of Sciences, Beijing 100101, China

10. Lester and Sue Smith Breast Center, Baylor College of Medicine, Houston, TX 77030, USA

11. Department of Molecular and Human Genetics, Baylor College of Medicine, Houston, TX 77030, USA

12. Key Laboratory of Genetic Evolution and Animal Models, Yunnan Key Laboratory of Molecular Biology of Domestic Animals, Kunming Institute of Zoology, Chinese Academy of Sciences, Kunming 650233, China

13. School of Life Sciences, Hubei University, Wuhan 430415, China

14. Department of Neurology, Henan Provincial People’s Hospital, People’s Hospital of Zhengzhou University, Zhengzhou, Henan 450003, China

15. China National Center for Bioinformation, Beijing 100101, China

16. Key Laboratory of Organ Regeneration and Reconstruction, State Key Laboratory of Membrane Biology, Institute of Zoology, Chinese Academy of Sciences, Beijing 100101, China

17. Institute for Stem cell and Regeneration, CAS, Beijing 100101, China

18. Aging Biomarker Consortium, Beijing 100101, China

19. Sino-Danish College, University of Chinese Academy of Sciences, Beijing 100049, China

20. Advanced Innovation Center for Human Brain Protection, and National Clinical Research Center for Geriatric Disorders, Xuanwu Hospital Capital Medical University, Beijing 100053, China

21. Aging Translational Medicine Center, Xuanwu Hospital, Capital Medical University, Beijing 100053, China

22. Chongqing Renji Hospital, University of Chinese Academy of Sciences, Chongqing 400062, China

23. State Key Laboratory of Stem Cell and Reproductive Biology, Institute of Zoology, Chinese Academy of Sciences, Beijing 100101, China

24. Beijing Institute for Stem Cell and Regenerative Medicine, Beijing 100101, China

25. State Key Laboratory of Membrane Biology, Institute of Zoology, Chinese Academy of Sciences, Beijing 100101, China

26. State Key Laboratory of Experimental Hematology, Institute of Hematology and Blood Diseases Hospital, Chinese Academy of Medical Sciences and Peking Union Medical College, Tianjin 300020, China

27. Department of Precision Medicine, Changhai Hospital, Second Military Medical University (Naval Medical University), Shanghai, 200433, China

28. School of Health Science and Engineering, University of Shanghai for Science and Technology, Shanghai, 200093, China

29. Institute of Artificial Intelligence, Donghua University, Shanghai 201620, China

30. Department of Hepatobiliary Pancreatic Surgery, Changhai Hospital, Second Military Medical University (Naval Medical University), Shanghai 200433, China

31. State Key Laboratory for Macromolecule Drugs and Large-scale Manufacturing, School of Pharmaceutical Sciences, Wenzhou Medical University, Wenzhou 325030, China

32. Department of Oncology, 905th Hospital of PLA Navy Naval Medical University, Shanghai 200433, China

33. Center for Translational Medicine, Second Military Medical University (Naval Medical University), Shanghai 200433, China

34. National Key Laboratory of Immunity and Inflammation, Institute of Immunology, Naval Medical University, Shanghai 200433, China

35. Department of Gastroenterology, Shanghai Tenth People’s Hospital, School of Life Sciences and Technology, Tongji University, Shanghai 200072, China

36. National Center, Children’s Hospital, Zhejiang University School of Medicine, National Clinical Research Center for Child Health, Hangzhou 310052, China

37. CAS Center for Excellence in Molecular Cell Science, Chinese Academy of Sciences, Shanghai 200031, China

38. Department of Biology, Southern University of Science and Technology, Shenzhen, Guangdong 518055, China

39. Key Laboratory of Synthetic Biology, Key Laboratory of Plant Design, CAS Center for Excellence in Molecular Plant Sciences, Institute of Plant Physiology and Ecology, Chinese Academy of Sciences, Shanghai 200031, China

40. State Key Laboratory of Microbial Metabolism, School of Life Sciences and Biotechnology, Shanghai Jiao Tong University, Shanghai 200240, China.

41. School of Oceanography, Shanghai Jiao Tong University, Shanghai 200240, China

42. Huazhong University of Science and Technology, Wuhan, Hubei 430074, China

43. Interdisciplinary Institute for Medical Engineering, Fuzhou University, Fuzhou 350002, China

44. College of Sericulture, Textile and Biomass Sciences, Southwest University, Chongqing 400715, China

45. State Key Laboratory of Plant Cell and Chromosome Engineering, Institute of Genetics and Developmental Biology, Chinese Academy of Sciences, Beijing 100101, China

46. Kunming College of Life Science, University of Chinese Academy of Sciences, Kunming, Yunnan 650204, China

47. State Key Laboratory for Conservation and Utilization of Bio-resources, Yunnan University, Kunming 650091, China

48. Yunnan Key Laboratory of Biodiversity Information, Kunming Institute of Zoology, Chinese Academy of Sciences, Kunming, Yunnan 650223, China

49. Experimental Research Center, China Academy of Traditional Chinese Medicine, Beijing 100700, China

50. State Key Laboratory for Quality Ensurance and Sustainable Use of Dao-di Herbs, China Academy of Chinese Medical Sciences, Beijing 100000, China

51. Biomedical big data center, the First Affiliated Hospital, Zhejiang University School of Medicine, Zhejiang 310003, China

52. Hubei Key Laboratory of Agricultural Bioinformatics, College of Informatics, Huazhong Agricultural University, Wuhan 430070, P.R. China

53. Department of Thoracic Surgery, West China Biomedical Big Data Center, West China Hospital, Sichuan University, Chengdu 610041, China

54. State Key Laboratory of Oncology in South China, Cancer Center, Collaborative Innovation Center for Cancer Medicine, School of Life Sciences, Sun Yat-sen University, Guangzhou 510060, China

55. College of Bioinformatics Science and Technology, Harbin Medical University, Harbin, Heilongjiang 150081, China

56. MOE Key Laboratory of Molecular Biophysics, Hubei Bioinformatics and Molecular Imaging Key Laboratory, College of Life Science and Technology, Huazhong University of Science and Technology, Wuhan, Hubei 430074, China

57. Beijing Neurosurgical Institute, Capital Medical University, Beijing 100070, China

58. Beijing Institutes of Life Science, Chinese Academy of Sciences, Beijing 100101, China

59. Key Laboratory of Systems Biology, Hangzhou Institute for Advanced Study, University of Chinese Academy of Sciences, Chinese Academy of Sciences, Hangzhou 310020, China

60. Department of Bioinformatics, College of Life Sciences; The First Affiliated Hospital, School of Medicine, Zhejiang University, Hangzhou 310058, China

61. Institute of Systems, Molecular and Integrative Biology, University of Liverpool, Liverpool L69 7ZB, Liverpool, UK

62. Department of Biological Sciences, Xi’an Jiaotong-Liverpool University, Suzhou, Jiangsu 215123, China

63. Department of Physics, School of Science, Tianjin University, Tianjin 300072, China

64. Frontiers Science Center for Synthetic Biology and Key Laboratory of Systems Bioengineering (Ministry of Education), Tianjin University, Tianjin 300072, China

65. SynBio Research Platform, Collaborative Innovation Center of Chemical Science and Engineering (Tianjin), Tianjin 300072, China

66. Key Laboratory of Gastrointestinal Cancer (Fujian Medical University), Ministry of Education, Fuzhou 350122, China

67. Fujian Key Laboratory of Tumor Microbiology, Department of Medical Microbiology, School of Basic Medical Sciences, Fujian Medical University, Fuzhou, Fujian 350004, China

68. Key Laboratory of Zoological Systematics and Evolution, Institute of Zoology, Chinese Academy of Sciences, Beijing 100101, China

69. Center for Stem Cell and Regenerative Medicine, Zhejiang University School of Medicine, Hangzhou 310058, China

70. State Key Laboratory for Quality Ensurance and Sustainable Use of Dao-di Herbs, National Resource Center for Chinese Materia Medica, China Academy of Chinese Medical Sciences, Beijing 100000, China

71. Department of Public Health, School of Medicine, Nanjing University of Chinese Medicine, Nanjing 210023, China

72. Computational Bioscience Research Center (CBRC), Computer, Electrical and Mathematical Sciences and Engineering Division, King Abdullah University of Science and Technology (KAUST), Thuwal 23955, Saudi Arabia

73. Hunan Provincial Key Lab on Bioinformatics, School of Computer Science and Engineering, Central South University, Changsha 410083, China

74. State Key Laboratory of Protein and Plant Gene Research, School of Life Sciences, Biomedical Pioneering Innovative Center (BIOPIC) & Beijing Advanced Innovation Center for Genomics (ICG), Center for Bioinformatics (CBI), Peking University, Beijing 100871, China

75. State Key Laboratory of Protein and Plant Gene Research, Laboratory of Bioinformatics and Genomic Medicine, Institute of Molecular Medicine, College of Future Technology, Peking University, Beijing 100080, China

76. Institute of Life Course and Medical Sciences, University of Liverpool, Liverpool L7 8TX, UK

77. Sun Yat-sen University Cancer Center, Guangzhou, Guangdong 510060, China

78. NHC Key Laboratory of Systems Biology of Pathogens, Institute of Pathogen Biology, Chinese Academy of Medical Sciences and Peking Union Medical College, Beijing, P.R. China.
